# SARS-CoV-2/COVID-19 and advances in developing potential therapeutics and vaccines to counter this emerging pandemic

**DOI:** 10.1186/s12941-020-00384-w

**Published:** 2020-09-02

**Authors:** Ali A. Rabaan, Shamsah H. Al-Ahmed, Ranjit Sah, Ruchi Tiwari, Mohd. Iqbal Yatoo, Shailesh Kumar Patel, Mamta Pathak, Yashpal Singh Malik, Kuldeep Dhama, Karam Pal Singh, D. Katterine Bonilla-Aldana, Shafiul Haque, Dayron F. Martinez-Pulgarin, Alfonso J. Rodriguez-Morales, Hakan Leblebicioglu

**Affiliations:** 1 Molecular Diagnostic Laboratory, Johns Hopkins Aramco Healthcare, Dhahran, Saudi Arabia; 2 Specialty Paediatric Medicine, Qatif Central Hospital, Qatif, Saudi Arabia; 3 Department of Microbiology, Tribhuvan University Teaching Hospital, Institute of Medicine, Kathmandu, Nepal; 4 Department of Veterinary Microbiology and Immunology, College of Veterinary Sciences, UP Pandit Deen Dayal Upadhayay Pashu Chikitsa Vigyan Vishwavidyalay Evum Go-Anusandhan Sansthan (DUVASU), Mathura, 281001 India; 5 Sher-E-Kashmir University of Agricultural Sciences and Technology of Kashmir, Shalimar, Srinagar, Jammu and Kashmir 190025 India; 6 Division of Pathology, ICAR-Indian Veterinary Research Institute, Izatnagar, Bareilly, Uttar Pradesh 243 122 India; 7 Division of Biological Standardization, ICAR-Indian Veterinary Research Institute, Izatnagar, Bareilly, Uttar Pradesh 243 122 India; 8 Semillero de Investigación en Zoonosis (SIZOO), Grupo de Investigación BIOECOS, Fundación Universitaria Autónoma de las Américas, Sede Pereira, Pereira, Risaralda Colombia; 9 Public Health and Infection Research Group, Faculty of Health Sciences, Universidad Tecnologica de Pereira, Pereira, Colombia; 10 Research and Scientific Studies Unit, College of Nursing & Allied Health Sciences, Jazan University, Jazan, Saudi Arabia; 11 Grupo de Investigación Biomedicina, Faculty of Medicine, Fundación Universitaria Autónoma de las Americas, Pereira, Risaralda Colombia; 12 School of Medicine, Universidad Privada Franz Tamayo (UNIFRANZ), Cochabamba, Bolivia; 13 Department of Infectious Diseases, Samsun VM Medicalpark Hospital, Samsun, Turkey

**Keywords:** SARS-CoV-2, COVID-19, Immunotherapeutics, Therapeutics, Vaccines

## Abstract

A novel coronavirus (SARS-CoV-2), causing an emerging coronavirus disease (COVID-19), first detected in Wuhan City, Hubei Province, China, which has taken a catastrophic turn with high toll rates in China and subsequently spreading across the globe. The rapid spread of this virus to more than 210 countries while affecting more than 25 million people and causing more than 843,000 human deaths, it has resulted in a pandemic situation in the world. The SARS-CoV-2 virus belongs to the genus *Betacoronavirus*, like MERS-CoV and SARS-CoV, all of which originated in bats. It is highly contagious, causing symptoms like fever, dyspnea, asthenia and pneumonia, thrombocytopenia, and the severely infected patients succumb to the disease. Coronaviruses (CoVs) among all known RNA viruses have the largest genomes ranging from 26 to 32 kb in length. Extensive research has been conducted to understand the molecular basis of the SARS-CoV-2 infection and evolution, develop effective therapeutics, antiviral drugs, and vaccines, and to design rapid and confirmatory viral diagnostics as well as adopt appropriate prevention and control strategies. To date, August 30, 2020, no effective, proven therapeutic antibodies or specific drugs, and vaccines have turned up. In this review article, we describe the underlying molecular organization and phylogenetic analysis of the coronaviruses, including the SARS-CoV-2, and recent advances in diagnosis and vaccine development in brief and focusing mainly on developing potential therapeutic options that can be explored to manage this pandemic virus infection, which would help in valid countering of COVID-19.

## Introduction

Since December 2019, a pneumonia-like Coronavirus emerged in Wuhan, Hubei province of China. Within a few weeks, a novel coronavirus was given a nomenclature as 2019 novel coronavirus (2019-nCoV) by the World Health Organization (WHO) and severe acute respiratory syndrome coronavirus-2 (SARS-CoV-2) by the International Committee on Taxonomy of Viruses (ICTV) [1–4], while the disease termed as an emerging coronavirus disease 2019 (COVID-19). Since then, this virus has spread to every populous continent and infected millions around the world [5]. After severely affecting few countries particularly such as China, Italy and Iran, the vast majority of confirmed cases, and fatalities presently fall within USA, Brazil, India, Russia, South Africa, Peru, Mexico, Colombia, Chile, Spain, UK, France, Germany [6, 7], and multiple countries in the globe. COVID-19 has now spread to more than 210 countries, with more than 25 million confirmed cases and nearly 843,000 human deaths (on August 30, 2020). WHO declared it a pandemic situation worldwide [8, 9]. Unlike other coronavirus epidemics like SARS and MERS (Table 1), the transmission rate of COVID-19 is much higher, alarming infection to an average of two to three individuals getting affected from one infected individual [10–13].

**Table 1:** Classification of Coronavirus [260]

Classification	Viruses
Alphacoronavirus	1. Alphacoronavirus 1 2. Porcine epidemic diarrhoea virus (PEDV) 3. Bat coronavirus 1 4. BtCoV 512 5. BtCoV-HKU8 6. BtCoV-HKU2 7. HCoV-NL63 8. HCoV-229E
Betacoronaviru (4 Lineages including A, B, C, D)	Lineage A: 1. HCoV-OC43 2. HCoV-HKU1, 3. Betacoronavirus 1 (more commonly known as bovine coronavirus, BCoV) 4. Murine coronavirus (MHV) Lineage B: 1. Severe acute respiratory syndrome-related SARS-CoV Lineage C: 1. *Tylonycteris* bat coronavirus HKU4 (BtCoV-HKU4) 2. *Pipistrellus* bat coronavirus HKU5 (BtCoV-HKU5) 3. Middle East Respiratory Syndrome MERS-CoV Lineage D: *Rousettus* bat coronavirus HKU9 (BtCoV-HKU9)
Gammacoronavirus	1. Avian coronavirus 2. Whale coronavirus SW1
Deltacoronavirus	1. HKU11 2. HKU12 3. HKU13

Travellers have been mainly implicated in giving wings to SARS-CoV-2 for its worldwide spread [5, 14, 15]. COVID-19 is spreading rapidly and crossing borders too. It has been stated that most of the affected patients in China worked at or lived in the Huanan seafood wholesale market, where live animals like snakes, bats, birds, marmots, and other wildlife farm animals were sold [16, 17]. Wuhan was considered a hotspot for emerging infectious diseases. The news of numerous cases of pneumonia was reported from December 31, 2019, with unknown aetiology in Wuhan, China [18], which was thought to be SARS-CoV (an epidemic which originated in China as well in 2002). Finally, it was confirmed that the SARS-CoV-2 virus, too, belongs to the family of viruses that include the common cold, and viruses of SARS and MERS [19–21]. The available yet limited epidemiological and clinical data for SARS-CoV-2 suggest that the disease spectrum and transmission efficiency of this virus differ from those reported for SARS-CoV [22, 23]. Apart from implications of SARS-CoV-2 to have its origin from bats and pangolins and then documented to affect humans leading to mainly respiratory infection and also to cause multiple system infection, the virus infection has also been reported from few animals such as cats, dogs, tiger, lion and mink [24]. Infected patients develop symptoms of high fever, dyspnea, pneumonia, chest pain, dry cough, myalgia and diarrhoea as clinical signs of the disease [25, 26] and few other non-respiratory illness symptoms too [27–30].

Structures of many individual proteins of SARS, MERS, and related coronaviruses, as well as their biological interactions with other viral and host proteins, have been explored along with the experimental testing of anti-viral properties of small compounds [31–33]. To date, there are no proclaimed clinically proven therapeutic antibodies, drugs, and vaccines specific for coronaviruses, which makes it tougher to tackle SARS-CoV-2. This article, in brief, describes the molecular organization and phylogenetic analysis of the coronaviruses, including the SARS-CoV-2 highlights few advances in diagnosis and vaccine development [34]. It elaborately emphasizes on the different potential therapeutic options that could be pursued for therapy despite limited knowledge of the biology of SARS-CoV-2 such as neutralizing antibodies, oligonucleotides, passive antibody transfer, and drug repurposing, anti-viral proteases, blocking Coronavirus receptors like an angiotensin-converting enzyme (ACE2), combination therapy, which can bring a revolutionary change to curb the SARS-CoV-2/COVID-19 in the coming future [35, 36].

## Phylogenetic analysis and genomic organization

Recent phylogenetic analysis has revealed that the new virus which commenced its spreading from China is a version of SARS-CoV[37, 38]. There are two significant similarities between SARS-CoV-2 (nCoV-2019) and SARS-CoV: they share nearly 80% of their genetic codes, and both were originated in bats [39, 40]. The first study to analyze the viral genome analysis was conducted by the Wuhan Institute of Virology (Table 2). Samples from seven patients initially complaining of severe pneumonia were used in the report. It was found that the genetic of SARS-CoV-2 was 79.5%, similar to SARS-CoV [41].

**Table 2:** Comparative genome size of SARS-CoV-2 and other viruses

Virus	Genome (nt)	Proteins
Picornavirus (Polio)	7500	15
Retrovirus (HIV)	9700	15
Flavivirus (Dengue)	10,700	10
Togavirus (Chikungunya)	11,200	9
Rhabdovirus (Rabies)	13,500	11
Paramyxovirus (Mumps)	15,000	9
Reovirus (Rotavirus)	18,500	12
Filovirus (Ebola)	19,000	8
*Coronavirus (SARS-CoV-2)*	*29,900*	*26*

The genome of CoVs consists, a single-stranded, positive-sense RNA of around 29.8 kb nucleotides in length with a 5′-cap structure and 3′-polyA tail. The polyprotein 1a/1ab (pp1a/pp1ab) is directly translated using genomic RNA as a template, which encodes nonstructural proteins (nsps) and forms the replication-transcription complex (RTC). Also, a nested set of subgenomic RNAs (sgRNAs) are produced by the replication-transcription complex in a discontinuous manner of transcription. It has common 5′-leader sequences and 3′-terminal sequences. Transcription termination and acquisition of leader RNA happens at transcription regulatory sequences, which are located between open reading frames (ORFs). These sgRNAs (minus-strand) acts as the templates for the synthesis of subgenomic mRNAs[18, 42, 43].

It has also been established that the genomes of SARS-CoV-2, SARS-CoV, and MERS-CoV strains are almost identical and possess only six nucleotide differences in the genome. A typical CoV’s genome and subgenome contain at least six ORFs. The first ORF (ORF1a/b) contains two-third of the whole length genome and encodes 16 nsps (nsp1-16). A-1 frameshift between ORF1b and ORF1a, leads to the synthesis of two polypeptides: pp1ab and pp1a. These are processed by chymotrypsin-like protease or two papain-like protease or main protease and one into 16 nsps. The rest of the ORFs encode four major structural proteins: membrane (M), spike (S), nucleocapsid (N), and envelope (E) proteins. Other than these proteins, different CoVs encode specific accessory proteins and structural proteins, such as 3a/b protein, HE protein, and 4a/b protein [18, 43].

The S protein (~ 150 kDa) mediates attachment of the virus to the host cell surface receptors resulting in the fusion and subsequent viral entry. The S protein consists of two functional subunits; S_1_ for binding with the receptor of the host cell and S_2_ for the fusion of viral and cellular membranes. Different coronaviruses use different domains present within the S_1_ subunit helps the virus to recognize and bind to specific receptors [18]. In some CoVs, the S protein also mediates cell–cell fusion between infected and adjacent, uninfected cells resulting in the formation of multinucleated giant cells. This strategy allows direct viral spread between cells while avoiding virus-neutralizing antibodies. The S protein utilizes an N-terminal signal sequence to gain access to the endoplasmic reticulum (ER) and is heavily N-linked glycosylated. Homotrimers of the virus-encoded S protein make up the distinctive spike-like structure [44, 45].

The M protein (~ 25–30 kDa) with three transmembrane domains is the most abundant structural protein and defines the shape of the viral envelope[46, 47]. Interaction of S with M protein is necessary for retention of S in the ER-Golgi intermediate compartment (ERGIC)/Golgi complex and its incorporation into new virions. However, it is not required for the assembly process[48].

The E protein (~ 8–12 kDa) is the smallest of the major structural proteins. This transmembrane protein has an N-terminal ectodomain and a C-terminal endodomain with ion channel activity. During the replication cycle, E is abundantly expressed inside the infected cell, but only a small portion is incorporated into the virus envelope[49].

The N protein is the only one that binds to the RNA genome (DST and Health Commission China, 2020). The protein is composed of two separate domains, an N-terminal domain (NTD) and a C-terminal domain (CTD). It has been suggested that optimal RNA binding requires a contribution from both these domains[50]. It is also involved in viral assembly and budding [51], resulting in complete virion formation.

The genomic sequence alignment among different CoVs shows 58% identity on the non-structural protein-coding region, 43% identity on the structural protein-coding region, with 54% at the whole genome level which suggests the non-structural proteins are more conserved when compared to the structural proteins and structural proteins are more diverse when in need of adaptation for new hosts. However, the CoV genome is extensive, with ~ 30 kb in length, and is considered as the largest known RNA viruses (Table 2). Such a large genome of CoVs is maintained by the unique features of the CoV RTC, which encodes for many RNA processing enzymes. The 3′–5′ exoribonuclease is one such kind of RNA processing enzyme and is unique in CoVs among different RNA viruses, also believed in providing a proofreading function to the RTC. Sequence analysis depicts that the SARS-CoV-2 has a typical genome structure the same as of CoV and mainly belongs to the *Betacoronaviruses* cluster, which includes Bat-SL ZXC21, Bat-SARS-like (SL)-ZC45, SARS-CoV, and MERS-CoV [18, 42]. The glycoprotein spike surface plays an essential role in receptor binding and determines host tropism [52]. These proteins of SARS-CoV and MERS-CoV bind via different receptor-binding domains (RBDs) to various host receptors. SARS-CoV utilizes angiotensin-converting enzyme 2 (ACE2) as the primary receptor, and MERS-CoV uses dipeptidyl peptidase 4 (DPP4, also known as CD26) as the primary receptor. Initial analysis depicted that SARS-CoV-2 has a quite close phylogenetic association with SARS-like bat coronaviruses [39, 53].

An observation of the amino acid substitutions in different proteins occurs among them, which sheds light on how SARS-CoV-2 differs from SARS-CoVs structurally and functionally. In total, 380 amino acid substitutions were there between the SARS-CoV-2 amino acid sequences and the SARS and SARS-like viruses. However, no amino acid substitutions were found in nonstructural protein 7 (nsp7), envelope, nsp13, matrix, or accessory proteins 8b and p6. Nevertheless, due to minimal knowledge about this novel virus, it is quite hard to give reasonable explanations for the amino acid substitutions between the SARS-CoV-2 and SARS or SARS-like CoVs and also whether these differences could affect the host transmission property of the SARS-CoV-2 when compared to SARS-CoV needs future investigation [54].

SARS-CoV-2 became the seventh member of the human coronaviruses (HCoVs) identified as HCoV-229E, HCoV-OC43, HCoV-NL63, HCoV-HKU1, SARS-CoV (which causes severe acute respiratory syndrome), MERS-CoV (Middle East respiratory syndrome), and now SARS-CoV-2 [55].

**Fig. 1:**
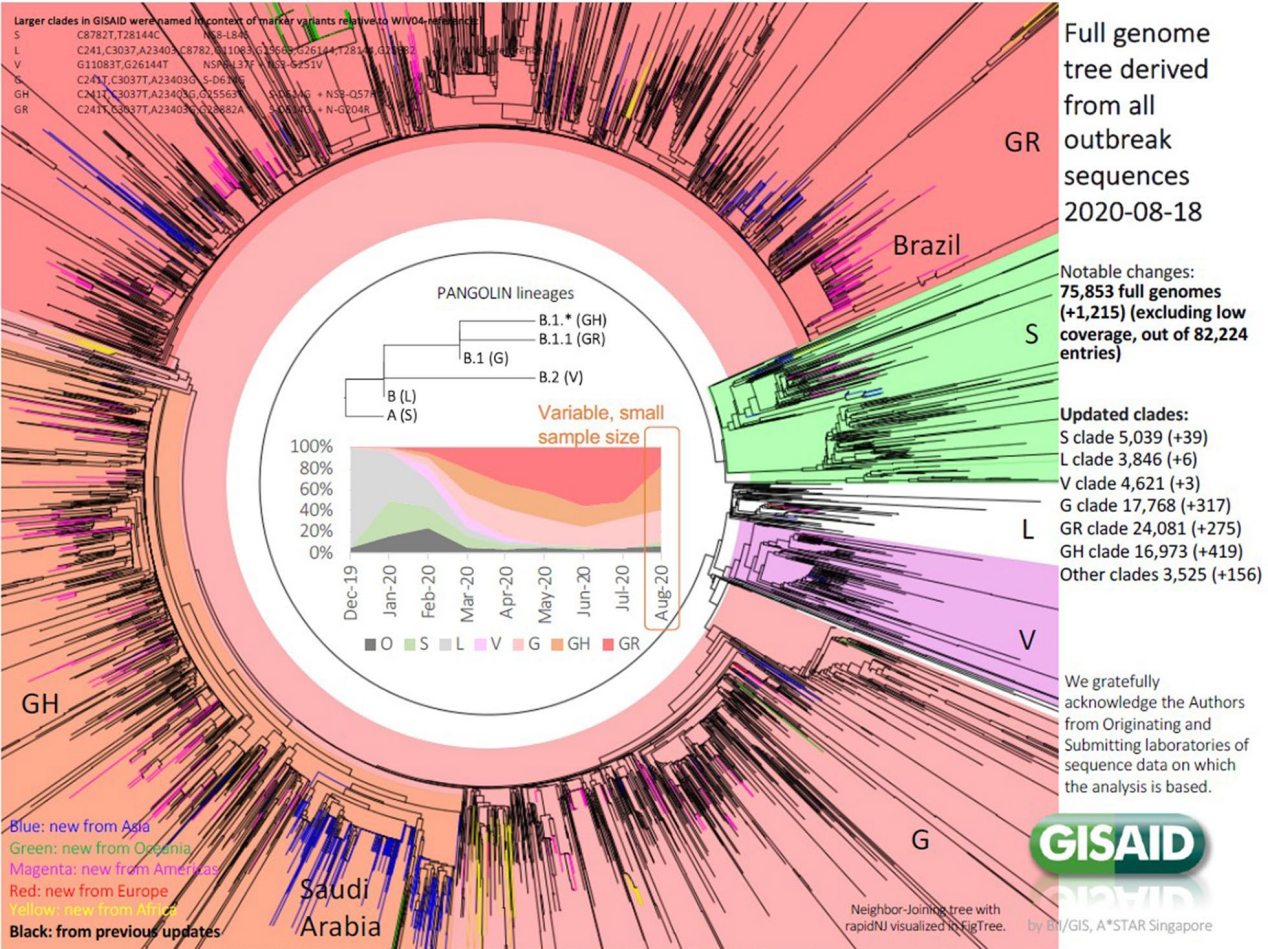
Genome sequence phylogenetic analyses of GISAID (http://www.gisaid.org)

Currently, the Global Initiative on Sharing All Influenza Data (GISAID) (http://www.gisaid.org), have analyzed over 75,853 full genomes of SARS-CoV-2 up to August 30, 2020, with the predominance of the GR clade (23,502), followed by the G clade (17,176), and the GH clade (16,416), among others (Fig. 1).

## The emergence of Coronaviruses (SARS-CoV, MERS CoV, and SARS-CoV-2)

The Coronaviruses were known to cause only mild respiratory disease in humans, with severe infections in the immunocompromised cases and young population mostly acting as an asymptomatic carrier because disease symptoms are not much prominent in young ones [56]. Coronaviruses have the highest known frequency of recombination of any positive-strand RNA virus [55]. Earlier reports suggested the emergence of SARS-CoV by recombination between bat SARS related coronaviruses (SARSr-CoVs) followed by mutations in civets before its spillover[57, 58]. Similarly, the MERS-CoV also circulated and attained mutations for around 30 years in camels before the MERS pandemic happened [59, 60], suggesting the adaptation of the viruses to the environment and different host before their spillover to humans [61]. Coronaviruses also have an essential replication process, which involves a 2-step replication mechanism. Many RNA virus genomes contain a single open reading frame (ORF), but coronaviruses can contain up to 10 separate ORFs except for SARS-CoV-2, which has single intact ORF [55]. That can lead to the emergence of CoV at times.

### SARS-CoV

SARS-CoV emerged first in the Guangdong Province of China; and spread internationally to 37 different countries. Noteworthy outbreak sites included China, Hong Kong, Taiwan, Singapore, and Vietnam. However, outside the Southeast Asia area, Canada was also significantly affected. The WHO reported 8,437 SARS cases and 813 deaths between the period of November 2002 to July 2003. Transmission is presumed to be from Chinese horseshoe bats harboring SARS-like viruses directly to humans or via intermediate hosts, i.e., palm civets or raccoon dogs in the live-animal markets of China. The employment of control measures inclusive of quarantine and air travel blockade[62], ultimately resulted in the disappearance of SARS-CoV in 2003, with no human infections reported ever since [21, 63]. The structural and genomic similarities between the two viruses have been discussed earlier.

### MERS-CoV

MERS-CoV was first identified in the Kingdom of Saudi Arabia in 2012. Cases have been geographically restricted to the Arabian Peninsula. However, a smaller number of cases were also found in Europe, i.e., in people who had travelled to the Arabian Peninsula or had been in contact with infected individuals. Individual cases and small clusters continue to be reported in that region. MERS-CoV is thought to be transmitted from camels to humans, with the possibility that at some point bats infected camels. MERS-CoV is thought to have emerged from bats or other small animals, and have infected humans via dromedary camels acting as intermediate hosts [20, 64].

### SARS-CoV-2

A novel (new) coronavirus (SARS-CoV-2) that was first detected in Wuhan City, Hubei Province, China, is posing a significant threat now by creating a pandemic situation of the COVID-19 [9, 65]. The first case was detected in December 2019, and presently above 22 million confirmed cases have been identified [8]. The disease terrorized over 210 countries/territories/areas with 785,000 deaths, which is probably not the last figure to be known [8]. In early March 2020, the pandemic started to subside in China [66] but began to haunt Europe and the United States, becoming Italy the epicentre of Europe and later New York city and state. In this context, other than China, the major blow was felt, in the first months of the pandemic, then with a significant impact at the UK, Italy, France, and Spain, in Europe. However, the increasing of cases and mortality was later higher in the US, where now there is the highest number of cases and deaths. Emerging situations, such as the significant increase in Russia, or the exponential growth in Brazil and other countries in Latin America, as well in India could not be overlooked [67].

Few countries are left beyond the reach of this unfortunate pandemic. However, their possibility of remaining untouched by this virus is meagre because, within a short period, the virus emerged in many new countries, including India, where the statistics are uprising[68]. Now the number of confirmed cases and deaths is shooting day by day with no hope of sudden halt, leaving the global population in a stage of physiological, psychological, and socioeconomic stress. In a study, the mental stress of the general public, along with nurses involved in the treatment of COVID-19 patients was evaluated based on vicarious traumatization scores and revealed that the scores were found significantly higher for the general public than nurses [69–71].

The origin of SARS-CoV-2 was postulated to be from bats, considering them the natural reservoirs of the virus [72]. The search for probable intermediate hosts is going on, keeping the main focus on animal species available for sale in the Huanan seafood market. Recently, a study suggested the pangolins to serve as a natural reservoir host of SARS-CoV-2-like coronaviruses (Pangolin-CoV) based on 91.02% sequence similarities at the whole-genome level between pangolin-CoV and SARS-CoV-2. Additionally, the S1 protein of pangolin coronavirus was reported to be closer to SARS-CoV-2 than bat coronavirus (RaTG13)[73]. Initially, the disease was thought to be spreading via seafood consumption, but later contact transmission of SARS-CoV-2 among humans was confirmed. In nearby places like clinics and hospitals, CoV can be transmitted through the air if people remain there for a long duration of time, suggesting the risk of aerosol transmission [74].

The emergence of SARS-CoV in 2002 established CoVs as capable of causing severe disease in humans. This ability was once again demonstrated by the emergence of a second severe CoV a decade later, the MERS-CoV in 2012. The MERS-CoV and SARS-CoV emergence are believed to be the result of spill-over of bat-adapted CoVs into an intermediate host. They are transmitted from person to person through respiratory droplets and close contact (https://www.cdc.gov/coronavirus/types.html). The virus is believed to be transmitted to patients living in or working at areas in proximity to wholesale seafood markets and places where live animals like snakes, bats, birds, marmots, and other wild or farm animals were sold [16]. Rarely animal coronaviruses infect humans severely except for SARS-CoV, MERS-CoV, and now SARS-CoV-2. Seldom coronaviruses cause lower respiratory infection or pneumonia but SARS-CoV-2 causes. Coronaviruses have seasonal pattern usually affecting in winter, however, can infect out of season also [55].

## Clinical pathology of SARS-CoV-2

The coronavirus claimed the first life on January 10, 2020, and since then, the death toll has climbed at an alarming and accelerating rate. The virus seems lethal, causing severe acute respiratory symptoms, including fever, dyspnea, asthenia and pneumonia, thrombocytopenia, and increased C-reactive protein and lactate dehydrogenase levels among people in Wuhan, China [40, 75]. As per reports, mild COVID-19 cases revealed higher levels of pro-inflammatory cytokines and chemokines like IFN-α, IL-1β, MCP-1, and IP-10 whereas individuals with severe COVID-19 had upregulation of IP-10, IL-8, IL-10, TNF-α, G-CSF, MCP-1 and MIP-1A [25, 76] resulting into cytokine storm syndrome followed by severe pulmonary damage and death due to respiratory failure. Additionally, all the blood cells except neutrophils were reported to be decreased with fall in lymphocytes subsets like T cells, B cells, and NK cells in severe COVID-19 cases [76]. Chest radiographs show invasive lesions in both lungs, with flaws of a variable degree in the lungs. Additionally, bilateral multilobular subsegmental consolidation, ground-glass opacity with many mottling was also reported in the COVID-19 patients [25, 26]. Recently, myalgia and fatigue are found associated with rhabdomyolysis in a COVID-19 patient in Wuhan, China suggesting the need for rapid clinical diagnosis followed by favourable hydration treatment to reduce the risk of severe outcomes as a result of rhabdomyolysis [70, 77].

Additionally, COVID-19 patients may also manifest neurological signs such as headache [29], nausea, and sometimes vomiting and diarrhoea [30], but even dysgeusia and anosmia [27, 78]. Moreover, SARS-CoV infection has been reported in the nervous tissue of experimental animals and patients with heavy involvement of the brainstem. In this context, acute respiratory failure among COVID-19 patients suggests the probable invasion of the brain by SARS-CoV-2 [79]. Recently, a study supported the neurotropic potential of the SARS-CoV-2 virus, as 36.4% of involved COVID-19 patients manifested neurological signs [80]. Those who developed severe pneumonia, pulmonary oedema, hypoxemic respiratory failure, gastrointestinal infection, multiple system failure, or Acute Respiratory Distress Syndrome (ARDS) succumb to the disease. The threat is still looming high in the event of this deadly virus created a pandemic situation [8, 9, 81–83]. Besides, there have been reports on renal failure, hepatic and pancreatic damage by SARS-CoV-2 [84, 85]. Destruction of microvasculature in various organs is believed to be one of the pathogenic mechanisms of the COVID-19 [84, 86].

## COVID-19 diagnostics

Several molecular diagnostics tests such as Real Time-PCR for genes encoding the internal RNA-dependent RNA polymerase and Spike’s receptor binding domain, full genome analysis by next-generation sequencing (NGS), isothermal loop-mediated amplification of coronavirus (i-LACO), multiplex nucleic acid amplification, and microarray-based assays are in use currently for the laboratory confirmation of coronavirus infection [87–89]. Full genome sequencing is the ultimate tool to study the origin and evolution of this novel virus, which has structural similarities to the earlier coronaviruses. That can also be helpful in guidance to the therapeutic outcome and genotyping.

Diagnostic tests for SARS-CoV-2/ COVID-19 cases include viral genome sequencing, RT-PCR, real-time RT-PCR (rRT-qPCR), POCT/bedside testing, reverse transcriptional loop-mediated isothermal amplification (RT-LAMP), serological assays (enzyme-linked immunoassay, ELISA), computed tomography technique (CT) imaging, X-ray [25, 88–92]. A reverse real-time PCR assay (rRT-PCR) is required for useful and timely screening of COVID-19 patients, which can be carried out in clinical samples like fibre bronchoscope brush biopsies, bronchoalveolar lavage, nasal swabs, pharyngeal swabs, sputum, blood and faeces [93]. An interactive web-based dashboard for monitoring COVID-19 in real-time mode has also been reported [94]. A fluorescence-based quantitative PCR assay based on SARS-CoV-2 N and ORF1ab regions has been developed [41]. Moreover, testing for COVID-19 requires travelling to a clinical setting that could lead to increased risk of disease transmission; hence a rapid, cheap, user-friendly, and sensitive diagnostic tool must be developed for use by ordinary people in their homes [95].In this context, a potential RNA-based POCT diagnostic device which combines a LAMP assay technology and a paper-based POCT was described as a home-based highly accessible and sensitive COVID-19 diagnostic tool with the additional advantage of smartphone integration enabling the individuals to record and share the results with healthcare workers and subsequent clinical care [95]. Currently, ELISA kits are being developed using the NGS data) to analyze the antigen presence. Recently a new ELISA kit was approved (Roche^®^). There are several quick tests for detection of IgM and IgG, but they were not extensively validated, and results are not reliable.

## COVID-19 immunotherapeutics and therapeutics

SARS-CoV-2 is now growing as a potential emerging pandemic [8, 9]. The lessons learned from earlier SARS-CoV-2 and MERS-CoV threats, recent and past epidemics and pandemics situations of Ebola, Zika, Nipah, swine flu, avian/bird flu led to make tremendous advances in science and research for developing suitable vaccines. Therapeutics/drugs, which along with the current research advances on COVID-19, are warranted to be explored optimally [19, 48, 91, 96–105]. The novel coronavirus is spreading rapidly from human to human with a wide range of clinical symptoms like fever, cough, myalgia, and fatigue with pneumonia [25, 40, 87]. To overcome the symptoms of novel coronavirus pneumonia, as per the severity of the patient, sedatives, analgesics, oxygen therapy, and ventilator facility should be provided [36, 106].

The current situation resembles the situation that happened with SARS in the 2002–2003 outbreak and the Ebola outbreak in 2014–2015 [107]. During this outbreak, special quarantine rules and protocols were set up to limit and identify the patient’s contact risk. There were no unique antiviral treatments available for SARS and Ebola at the time of outbreaks as situations were beyond control, similar to the SARS-CoV-2 outbreak. As the structure of the virus is known, so to prevent the virus entry and replication within the body of the host, various inhibitors producing hurdles at different steps are explored and tested in cell-based systems [108, 109]. They involve spike (S) protein inhibitors, S cleavage inhibitors, helicase and protease inhibitors, fusion core blockers, mAbs against host cell receptor, anti-viral peptide targeting S2, RBD–ACE2 blockers, antiviral peptides, siRNAs, neutralizing antibodies, convalescent-phase plasma, repurposed drugs, among others [47, 51, 110–112]. During the current outbreak and pandemic situation, patients require immediate treatment. The below sections will emphasize on different potential treatment options that could be pursued for therapy despite limited knowledge of the biology of SARS-CoV-2. Food and Drug Development Agency (FDA) has started Coronavirus Treatment Acceleration Program (CTAP), and presently there are more than 570 drug development programmes in planning stages, 270 plus trials reviewed, two treatments currently authorized for emergency use however no treatment is yet approved by FDA [113]. Of these treatments under investigation, more than 20 are based on antivirals, cell and gene therapies, 90 plus are based on immunomodulators, 30 plus on neutralizing antibodies, more than 70 include other treatments when the 30 plus are of combinations [113]. More than 210 have reached late stages when the 60 plus are at early stages of drug trials [113]. The use of chloroquine/hydroxychloroquine has also been suggested [114], though with some risk of harm. Besides, the evidence to support the use of lopinavir/ritonavir and remdesivir have also come up for treating COVID-19 patients [115].

### Developing neutralizing antibodies

In general, the infection of coronavirus starts with the entry of S protein, which binds to the surface of the cells. This S protein fuses with the cell membrane and helps the syncytial formation and delivering of viral nucleocapsids into the cell for further replication [116] and as per reports, neutralizing antibodies against receptor-binding domain (RBD) of S protein of SARS-CoV [117] and MERS-CoV [45, 118, 119] successfully neutralized those infections. In this context, neutralizing antibodies may prove highly useful in treating the COVID-19 as long as the SARS-CoV-2 shares the sequences in the RBD with SARS-CoV and MERS-COV [120].

For the time being, immunoglobulin G has been administered in COVID-19 critical patients as therapy [121, 122]. FcR has a role in pulmonary inflammation; hence blocking of FcR activation can reduce inflammatory damage in COVID-19. Thus intravenous use of immunoglobulins can prove helpful in the therapy of SARS-CoV-2 induced pulmonary inflammation [123].

S protein was targeted for developing a neutralizing antibody therapy to combat the new coronavirus disease [124]. Methods such as phage or yeast display libraries which express antibody fragments could be used efficiently to identify the candidate neutralizing antibody. Traditional methods of screening, such as mice or rabbits for neutralizing antibodies, would be too late during outbreaks. The only challenge is that neutralizing antibodies should be rigorously tested in animal and cell culture models to confirm that they can neutralize the SARS-CoV-2 disease infection [47, 125, 126]. The alternate strategy of generating the neutralizing antibodies against S protein is to immunize large animals like sheep, goat, cow, and horse and purify the polyclonal antibodies from these animals. Monoclonal antibodies can be used as potent bio-therapeutics in the form of passive immunotherapy to neutralize the SARS-CoV-2 and to control the harmful outcomes of COVID-19 [127]. These strategies may prove to be beneficial in the condition of an outbreak since they have many advantages, such as simplifying production and manufacturing. For a shorter treatment strategy, this could quickly help in the SARS-CoV-2 outbreak. Though antibody-based therapy is effective and immediate in use, it is short-lived. It has limitations of infection transmission, abnormal reactions, and the possibility of other severe risks like induction of severe acute lung injury or antibody-dependent enhancement [35, 127].

### Oligonucleotides targeting SARS-CoV-2 RNA genome

Apart from targeting S-protein of the nCoV-2019 virus using neutralizing antibodies, targeting of viral genomes could be another option to reduce the infectivity by degrading its genome. Recently, the RNA genome of the nCoV-2019 virus has been published (Gen Bank: MN908947.3). GS-5734, a nucleotide prodrug, showed broad-spectrum anti-coronavirus activity against bat CoV, pre-pandemic bat CoV, and existing human CoV in vitro and over primary human lung epithelial cell cultures and was found promising for treating epidemic and zoonotic coronaviruses of the near future [128]. The siRNA or antisense oligonucleotides (ASO) can be used to combat the virus by targeting its genome [129].

Nevertheless, there are a few challenges associated with these methods, such as conserved RNA sequence in the genome of coronaviruses is still not known. Since the conserved sequences are essential in siRNA targeting to avoid viral escape from the oligonucleotides targeting strategy. Second, the delivery of oligonucleotides (siRNA and ASO) would be very challenging. Lipid nanoparticle technology can mediate the delivery of these oligonucleotides into the lungs [130]. Even though they were successful in the preclinical studies in animal models [131, 132], siRNA candidates in viral infections like Ebola have failed in clinical trials [133]. Oligonucleoside analogues like remdesivir, which is a broad-spectrum antiviral drug, is proposed to have beneficial effects in COVID-19 therapy [111, 134]. Remdesivir and chloroquine have been reported to inhibit SARS-CoV-2 in vitro [35] effectively. They were able to block virus infection at low concentrations (micromolar) and showed a high selectivity index [135, 136].

However, these drugs have not proved enough efficacy in clinical trials. In the case of chloroquine and hydroxychloroquine, although initially was thought to be useful [114], clinical trials have shown no significant benefits, but significant adverse events associated [137–139]. There is insufficient evidence to support the effectiveness or safety of hydroxychloroquine or chloroquine for the treatment of COVID-19 in hospitalized patients as a systematic living review reported [140, 141]. In the case of remdesivir, appears to decrease the time to recovery by 2.5 days, but its clinical utility remains to be confirmed [142–144]. Remdesivir, chloroquine and hydroxychloroquine though being used in COVID-19 patients under the common emergencies but need to be appropriately evaluated for clinical applications in COVID-19 patients and side effects need to be explored for the safety purposes [145–148]. For dexamethasone, considered a boon for COVID-19 patients [149, 150] but a trial showed that it reduces mortality only in patients that require oxygen [151–153].

Further it has immunosuppressive effects also. Hence repurposing of these drugs with the proper formulation is required to improve their safety and effectiveness for the treatment of COVID-19 [154]. Alpha-lipoic acid, baloxavir, colchicine, interferon, lopinavir/ritonavir, favipiravir, ribavirin, ruxolitinib, among others, do not have RCTs, then no conclusions yet can be made [155]. Ribavirin and favipiravir are RNA polymerase inhibitors and have been used alone or in combination with IFN-α in COVID-19 patients [156]. Tocilizumab, ivermectin does not have RCTs yet, although they have preclinical data suggesting usefulness in COVID-19 [157, 158]. In any case, it should be considered that most of the drugs with biological plausibility and favourable preclinical studies ultimately did not have enough clinical benefits to allow their approval for commercialization. This indicates that medicines with biological plausibility and preclinical studies will often only cause harm and can even be fatal. Then, evidence-based decisions should be carefully assessed [159]. LAM-002A 9 (apilimod), a drug used to treat follicular lymphoma and autoimmune diseases have proven effective against SAR-CoV-2. Yale University and AI Therapeutics firm has started a phase II trial for LAM-002A 9 to assess its effectiveness for inhibiting SARS-CoV-2 (https://www.medicalnewstoday.com/articles/list-of-promising-drugs-against-covid-19-leads-to-new-treatment-trial).

### Passive antibody transfer

One of the most effective and traditional tools used in most of the infectious outbreaks is the use of serum of patients who just recovered from the active viral infection to treat patients who contract in the future [160]. Patients recovered from active viral infections develop a polyclonal immune response to different antigens of SARS-CoV-2 as they neutralize active viral infections. Hence, convalescent-phase plasma can be used as a therapeutic alternative [110, 161]. Passive immunotherapy in the form of convalescent serum tested in MERS-CoV infected mice either as prophylactic or in the therapeutic way both showed good results and supported the use of dromedary immune serum in preventing MERS-CoV infection [162]. Patients who have 100% recovery from the novel coronavirus infection, can simply donate their plasma to treat the infected patients [160, 163]. The same strategy of convalescent serum was used during the Ebola virus outbreak in 2014–2015 [164]. Plasma-derived from the patients who recovered from the disease has also been used as therapy [165]. It is the earliest and available means of treatment and provides antibodies that help in neutralizing SARS-CoV-2 however in addition to scarcity; this therapy may be non-specific and short-lived [35, 166] latter can be overcome by developing monoclonal antibodies [35, 127]. α-interferon atomization inhalation has been weakly recommended at a dose of 5 million U per time for adults in sterile injection water, twice a day [165]. Similarly, interferon therapy has also been used; however, it aggravated pathology [135].

### Drug repurposing using available antivirals

Drug repurposing is a promising, fast, and cost-effective method that can overcome traditional de novo drug discovery and development challenges of targeting various diseases and disorders. Drug repurposing, the process of identifying new uses for the existing or candidate drugs, is an effective strategy for drug discovery in multiple diseases, including infectious viral diseases. In combating the nCoV-2019 viral outbreak, the use of already approved small drug molecules could inhibit the biological aspects of the viral life cycle like replication, transcriptions, host protein interaction, boosting immunity, among others. Viral polymerase and protease inhibitors of HIV and hepatitis C virus are two potential antiviral regimens that can be repurposed against 2019-nCoV [167, 168]. During the SARS outbreak, HIV protease inhibitors like lopinavir and ritonavir had positive efficacy [169]. In vitro and in vivo (over rhesus macaque) experiments suggested that combined therapeutic regimen of lopinavir/ritonavir alone or in combination with recombinant interferon-β1b (IFN-β1b) have been found useful in treating MERS-CoV infection [110, 170, 171]. Hence, these are being explored as repurposed drugs against SARS-CoV-2 to address COVID-19 [172]. The study demonstrated that a combination of remdesivir (RDV) and interferon beta (IFN β) had better anti-viral efficacy as compared to the combined formula of lopinavir and ritonavir in treating MERS-CoV infections [173].

Since the novel coronavirus belongs to the same category of SARS, repurposing of HIV drugs against novel coronavirus could give positive efficacy. Lopinavir may have some prospects in COVID-19 therapy as has been in the SARS and MERS treatment; however, an extensive evaluation is required [174]. Efficacy of remdesivir against SARS-CoV-2 is under testing by Gilead Sciences (NASDAQ-GILD) pharmaceutical company (https://www.fool.com/investing/2020/03/04/is-gilead-sciences-the-best-buy-in-the-coronavirus.aspx). National Health Commission of the People’s Republic of China has advocated the inclusion of chloroquine phosphate for the cure of COVID-19 patients, in its revised guidelines for the prevention, diagnosis, and treatment of pneumonia developed due to COVID-19 infection in the vast populous country [175, 176]. Future research could continue to screen currently clinically available small molecular antiviral drugs in tissue culture models to identify candidate drugs to combat the novel coronavirus infection.

The pangolin CoV and SARS-CoV-2 are more than 92% similar at amino acid levels. Thus, using pangolin coronavirus as a model, three drugs, namely, cepharanthine (CEP), selamectin, and mefloquine hydrochloride, were observed to possess anti-SARS-CoV-2 activity with complete blocking of cell cytopathic effects [177].

Chen et al. [18] have used oseltamivir (75 mg), lopinavir (500 mg), ritonavir (500 mg) per os twice daily followed by ganciclovir (0.25 g) intravenously for 3–14 days in COVID-19 infected patients and they have shown safety hence can be considered as treatment options [18]. EIDD-2801 compound has proven effective against influenza viruses and can be evaluated against SARS-CoV-2 as well [178].

### Anti-viral proteases

Anti-coronavirus protease activity was exhibited by the lopinavir (LPV), and it is proposed as a treatment option for ongoing COVID-19 infection [174]. Further, a novel vinylsulfone protease inhibitor suggested treating patients suffering from the 2019-nCoV. That would help in the development of broad-spectrum anti-coronaviral agents for future epidemics [179]. Recently, a breakthrough in search of antivirals came with the elucidation of the SARS-CoV-2 main protease (Mpro) structure. The same could be exploited globally to design some novel drug candidates. Lately, a Deep Docking (DD) platform was used for structure-based virtual screening of nearly 1.3 billion molecules with the potential of 1,000 putative ligands for SARS-CoV-2 Mpro protein [180].

### Blocking Coronavirus receptors like ACE2

It is already known that ACE2 is a crucial player in the coronavirus infection by promoting cell entry [181]. The metallopeptidase, ACE2, has been identified as a functional receptor for SARS-CoV [182]and a potent receptor for SARS-CoV-2 [53]. The B domain of S_1_ in SARS-CoV-2 engages human ACE2 (hACE2) with comparable affinity found in SARS-CoV SB from viral isolates associated with the 2002–2003 epidemic (i.e., binding with high affinity to hACE2). The tight binding to hACE2 could partially explain the efficient transmission of SARS-CoV-2 in humans, as was the case for SARS-CoV [183]. ACE2 is an important drug target for the treatment of cardiovascular and kidney diseases [85, 184]. Recently, Lei et al. [185] demonstrated the potential of ACE2 based therapeutics against SARS-CoV-2, which could further be exploited alone or in combination, and they elucidate the molecular mechanisms of their potent and broad neutralizing activity. These ACE2 fusion proteins could be used for diagnosis and as research reagents in the development of vaccines and inhibitors. ACE2 and AT1R (angiotensin receptor one blocker) molecules such as losartan as inhibitors of the renin-angiotensin system (RAS) could be a useful therapeutic option in reducing the lung inflammation and treating pneumonia in COVID-19 patients [186, 187]. The virus attachment through spike glycoprotein (S) to ACE2 receptors and subsequent priming of S protein by the host cell serine protease TMPRSS2 has been exploited as therapeutic targets. In this context, the role of TMPRSS2 protease in SARS-CoV-2 replication has been reported, further supporting their probable role in the development of an active therapeutic agent [188]. The TMPRSS2 inhibitor proved useful in blocking the virus entry and could work as a therapeutic option [181].

### Combination therapy

Rothan and Byrareddy[135]have recommended the application of broad-spectrum antivirals like lopinavir/ritonavir, neuraminidase inhibitors, peptide (EK1), RNA synthesis inhibitors for the time being till specific antivirals are evaluated. Chen et al. [18] have used oseltamivir, lopinavir, ritonavir, ganciclovir in COVID-19 infected patients with excellent results; however, the combination of antiviral drugs is believed to be controversial [165]. Other medications used are antibiotics (cephalosporins, quinolones, carbapenems, tigecycline against methicillin-resistant *Staphylococcus aureus*, linezolid, moxifloxacin or levofloxacin, azithromycin or amoxicillin, and antifungal drugs), corticosteroids (prednisolone, dexamethasone), antipyretics (ibuprofen), anticoagulants (heparin) [18, 135, 165, 189, 190]. Huang et al. [25] used antibiotics, methylprednisolone corticosteroid (40–120 mg per day), and oseltamivir (orally 75 mg twice daily) in COVID-19 patients along with oxygen support. However, the use of antibiotics should be taken care of as patients may not always develop bacterial complications [50]. Other trials involved the use of lopinavir–ritonavir and interferon-α 2b in COVID-19 patients [191, 192]. National Health Commission of China recommended a combination of ribavirin and interferon-α as a treatment regimen for COVID-19 in its fifth edition; however, the efficacy of remdisivir and ritonavir/lopinavir needs to be determined by randomized controlled trial [50]. Rigorous preclinical and clinical both kinds of trials are required before the commencement of the commercialization of combination therapy against COVID-19 [193].

**Fig. 2:**
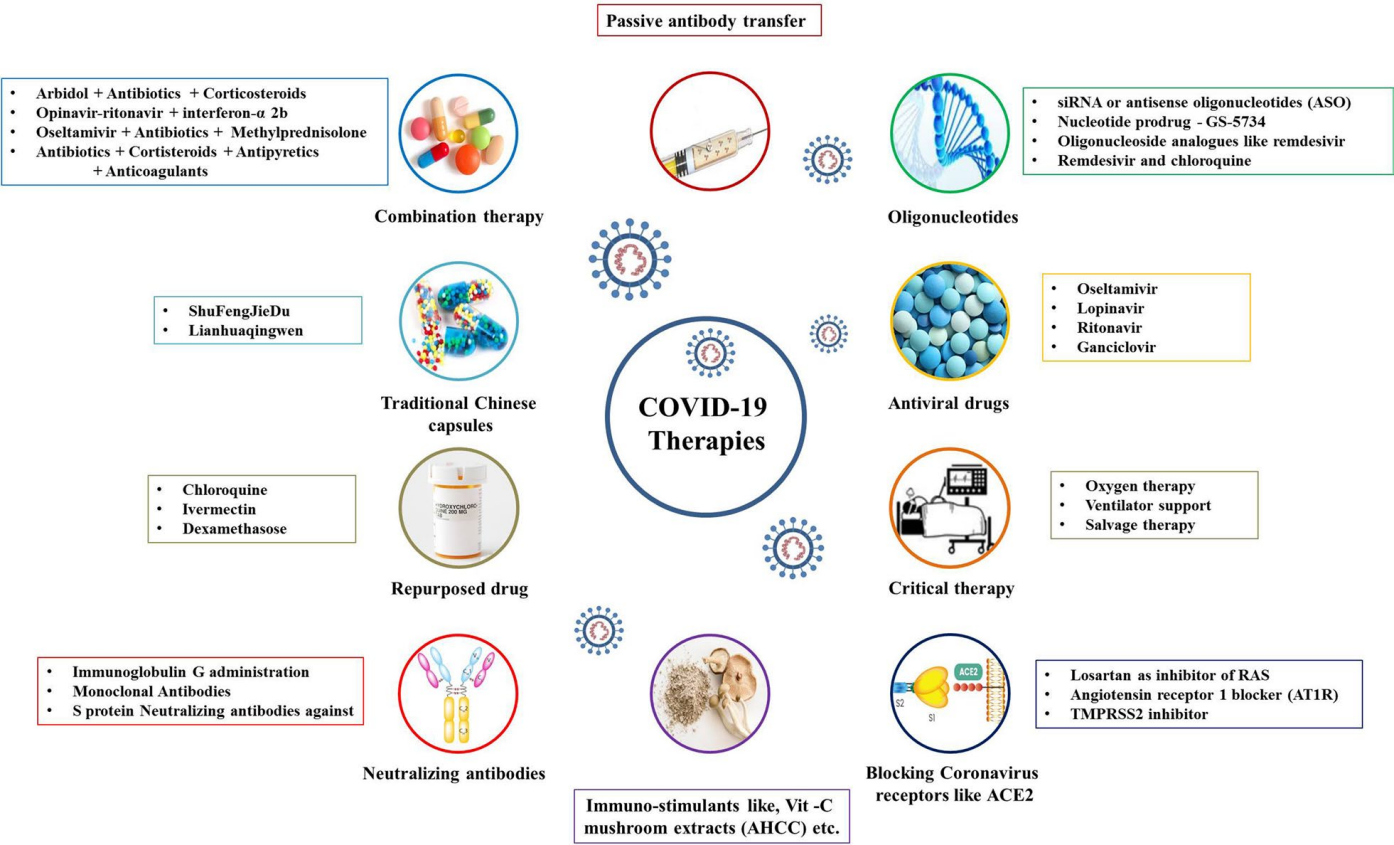
An overview of COVID-19 therapeutics and drugs

Arbidol is a broad-spectrum antiviral drug and blocks the fusion of the virus with the host cell [156], It is an effective antiviral against SARS-CoV in combination with antibiotics (moxifloxacin or levofloxacin, nemonoxacin, linezolid, azithromycin or amoxicillin), corticosteroids and oxygen therapy has been used in COVID-19 treatment [189]. Corticosteroids have been routinely used for the treatment of Th1 and Th2 induced lung injury reported in COVID-19 [25]. Conventional Chinese drugs like ShuFengJieDu and Lianhuaqingwen were also used in the treatment of COVID-19, but their efficacy needs to be determined [111]. In contrast to this, WHO has indicated that currently no effective treatment for SARS-CoV-2 is known and use of different antibiotics, antiviral drugs, traditional Chinese drugs, corticosteroids like glucocorticoid and their combinations are not recommended before clinical trials as their efficacy is not known and might be detrimental to COVID-19 patients [65, 194]. Besides, to manage the hypoxia in the COVID-19 patients’ ventilation and salvage therapy are reported beneficial. An α-glucan-based mushroom extract, i.e., AHCC has been reported to be used as an immunostimulant for animals and humans affected by viral infections like the influenza virus, West Nile virus, hepatitis virus, herpes virus, papillomavirus, and HIV. In this context, AHCC may be used in the prevention of COVID-19 after evaluation of its efficacy against SARS-CoV-2 [49]. For safe and successful treatment of severe respiratory illness in infants and children, the probability of atelectasis due to invasive or non-invasive ventilation support and risks of oxygen toxicity must be taken into account [195]. So far, until August 12, 2020, no effective therapy has been demonstrated in any study, including clinical trials. It seems that the most promising candidates, including as mentioned combination therapies, including remdesivir, and ivermectin [115]. Many newer drugs or candidates are being evaluated for application in COVID-19 patients. Teicoplanin, a glycopeptide antibiotic against staphylococci [196], has been recommended in complicated infections of COVID-19 and *Staphylococcus aureus* [197, 198]. It has proven effective in treating clinical conditions of patients. Danoprevir, a viral protease inhibitor, has also been used in a clinical trial on COVID-19 patients and showed considerable improvement in the situation of patients [199]. An overview of developing COVID-19 therapeutics and drugs is illustrated in Fig. 2.

## COVID-19 vaccines

**Fig. 3:**
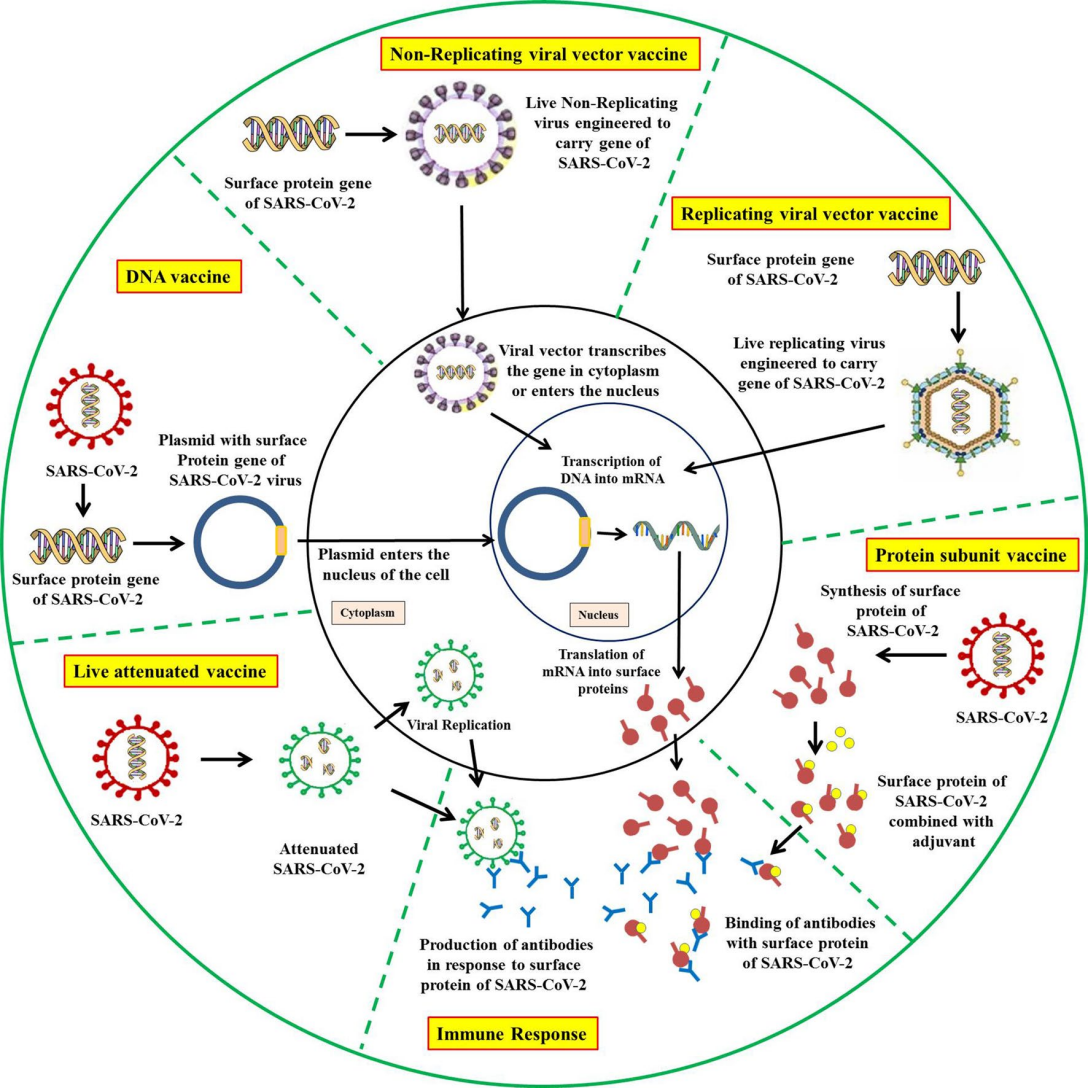
An overview of designing and developing COVID-19 vaccines

Currently, there are no specific vaccines available against COVID-19, but there are many candidates under development [192, 200–202]. Attempts are being made for the development of safe and effective prophylactic strategies [201, 203]. The earliest possibility is convalescent sera from the persons who recovered from the COVID-19 attack, which can be used as an immediate therapy (Fig. 3) [204].

In search of suitable vaccine against SARS-CoV-2, mRNA-based vaccine (mRNA-1273) by Moderna (NASDAQ: MRNA), INO-4800 by Inovio pharmaceuticals (NASDAQ-INO) and vaccines by other company such as CanSino Biologics, Sinovac Biotech Ltd., Johnson & Johnson, among others, are being developed and they have reached final stages of clinical trials [47, 205]. Moreover, a joint effort is made by Oxford University and Rocky Mountain Laboratories to develop a chimpanzee adenovirus vectored vaccine (ChadOx1)[206].

There are 29 vaccine candidates under clinical evaluation and 138 under preclinical evaluation as on August 13, 2020 [207, 208]. The ChAdOx1 encoded SARS-CoV-2 antigens vaccine with a non-replicating virus may trigger a robust immune response against the virus with the added advantage of safe immunization of geriatric people, children and individuals with comorbidities [209]. The ChAdOx1-S vaccine has shown that this adenovirus-vectored vaccine is immunogenic in mice, eliciting a robust humoral and cell-mediated response. This response is predominantly Th1, as demonstrated by IgG subclass and cytokine expression profiling. Vaccination with ChAdOx1 nCoV-19 (prime-only and prime-boost regimen) induces a balanced Th1/Th2 humoral and cellular immune response in rhesus macaques. Data showed that a significantly reduced viral load in bronchoalveolar lavage fluid and lower respiratory tract tissue of vaccinated rhesus macaques challenged with SARS-CoV-2 compared with control animals, and no pneumonia was observed in vaccinated animals. However, there was no difference in nasal shedding between vaccinated and control animals. Importantly, no evidence of immune-enhanced disease following viral challenge in vaccinated animals was observed. Safety, immunogenicity and efficacy of ChAdOx1 nCoV-19 against symptomatic PCR-positive COVID-19 illness will now be assessed in randomised controlled human clinical trials [210]. A preliminary report of a phase 1/2, single-blind, randomised controlled trial to evaluate the safety and immunogenicity of the ChAdOx1 nCoV-19 vaccine against SARS-CoV-2 have shown an acceptable safety profile, and homologous boosting increased antibody responses. These results, together with the induction of both humoral and cellular immune responses, support large-scale evaluation of this candidate vaccine in an ongoing phase 3 programme (ISRCTN89951424) [211].

A vaccine manufactured by Moderna Therapeutics in collaboration with NIAID has also completed clinical trials [212]. Additionally, a vaccine candidate expressing S protein of SARS-CoV-2 using mRNA vaccine platform technology is also in final stages [206]. Targeting spike proteins of CoV may have a role in vaccines and therapeutics as they induce highly potent neutralizing antibodies and are involved in host receptor binding and pathogenesis [213, 214]. Targeting spike glycoprotein (S glycoprotein) in SARS-CoV-2 can be useful [192]. However, multiple efforts to develop vaccines against SARS-CoV-2 have been made but not reach a commercial level and could excel only to the pre-clinical levels until the preparation of the manuscript. Recently, WHO released a list of 138 vaccine candidates under pre-clinical level grouped into five major groups like live attenuated virus vaccines, DNA vaccines, non-replicating viral vector vaccines, replicating viral vector vaccines, and subunit vaccines [215].

A DNA plasmid-based vaccine targeting S protein of the SARS-CoV-2 (INO-4800) has been developed by INOVIO Pharmaceuticals and reported to induce immune cells activation via intradermal inoculation [209, 216]. Moreover, the generation of cell-mediated and humoral immune responses in mice and guinea pigs were reported post-immunization with INO-4800 during pre-clinical studies along with the induction of specific neutralizing antibodies [216]. The phase 1 clinical trial of the INO-4800 reported its safety and well-tolerability in all volunteers along with stimulation of immune response in 94% of the volunteers against the SARS-CoV-2 after completion of phase 1 clinical trial [217].

The other modes of vaccine development are the utilization of either the virus itself or its part for developing whole organism-based vaccines or subunit vaccines. These include attenuated or inactivated vaccines using cultured SARS-CoV-2 that can be mitigated by passaging or inactivated by physical and chemical methods such as UV light, formaldehyde, and β-propiolactone [201]. However, these may have limitations of infectivity, reversion to pathogenicity, and disease-causing potential [218].

Exploration of vaccine candidates of SARS-CoV-2 is essential for the development of specific vaccines [192, 201], and the research and development are already being initiated [215]. A set of epitopes of SARS-CoV-2 have been screened, immune targeting of these epitopes can protect against this novel coronavirus and hence can provide experimental platforms for the development of vaccines [200]. Identification of putative protective antigen/peptide from SARS-CoV-2 is essential for the development of subunit vaccines [201]. The timely revealing of genome sequences is proving beneficial for subunit vaccine development [72, 219]. Structural proteins of SARS-CoV-2, including envelope (E), membrane (M), nucleocapsid (N), and spike (S), are being explored as antigens for subunit vaccine development [72, 201]. Ahmed et al. [200] have examined a set of T and B cell epitopes derived from Spike (S) and nucleocapsid (N) proteins mapping identically to SARS-CoV-2, and no mutations have been noted in these epitopes in 120 genome sequences hence can serve as vaccine candidates for the development of subunit vaccines.

Recently, antigenicity, along with structure and function of spike glycoprotein, especially of linear epitope S2 subunit of SARS-CoV-2 has been evaluated [183]. Neutralizing antibodies have been raised against the S2 subunit of SARS-CoV-2 that cross-react and neutralize both SARS-CoV-2 and SARS-CoV [183] hence, can be explored as candidates for subunit vaccines. Subunit vaccines have limitations of low immunity, the requirement of adjuvants, and sometimes inefficient protective immunity. In contrast, DNA and mRNA based vaccines are more accessible and quick to clinical trials, recombinant vaccines [201]. Viral-vector based vaccines can be constructed and used without adjuvant. These vaccines are possible only when the antigens having neutralizing epitopes are explored [201]. Mutated SARS-CoV-2, especially with altered E protein, can be exploited as a recombinant vaccine as N protein is conserved across CoVs hence not a suitable vaccine candidate [81, 201].

The vaccine based on the S2 protein subunit of the spike glycoprotein (S) that helps in receptor binding and entry can have broad-spectrum antiviral effects as it is conserved in SARS-CoV-2 [72, 81, 201]. Targeting S1 protein of SARS-CoV-2 can prevent virus entry and hence a strategy for controlling viral infection [201]. Targeting S protein can both develop both cellular and humoral immunity by inducing neutralizing antibodies and by developing protective cellular immunity [201]. Full-length S protein [220], receptor-binding domain (RBD) [221] of SARS-CoV have shown vaccine potential and can be explored in SARS-CoV-2 [201]. Similarly, the S1 subunit of spike protein in SARS-CoV-2 can be studied for antibody production hence prophylactic and therapeutic target [72]. Both aerosol or oral routes need to be explored as possible modes of administration [201].

DNA vaccines, chimeric viral vaccines, and membrane vesicle-vaccines are valuable options [201]. An mRNA-based vaccine by Moderna^®^ is believed to develop antibodies against spike proteins of SARS-CoV-2, and a batch of vaccine has been delivered to the National Institute of Allergy and Infectious Diseases. That was developed 42 days after the DNA sequence of SARS-CoV-2 was disclosed. Eli Lilly^®^ and AbCellera^®^ are evaluating Antibody-based treatment. GlaxoSmithKline^®^ has produced a pandemic vaccine adjuvant platform that it is partnering with other institutes and companies for use in COVID-19 vaccine candidates. Concerning this, recombinant DNA vaccines using GSK’s adjuvant are being developed by Sanofi^®^ in collaboration with GlaxoSmithKline (GSK) and expected to start phase 1/2 clinical study in September 2020 [222, 223].

The speed of COVID-19 vaccine development under the influence of pandemic is remarkable as only in six months from the first release of SARS-CoV-2 sequences the vaccines enter clinical trials. In contrast, a typical of 3 to 9 years are required for the same [224]. In context to this, the rapid pace is mainly attributed to the prior knowledge of S protein and its role in humoral immunity and coronavirus pathogenesis [225, 226]the evolution of multiple vaccine platforms and advanced activities in the process of vaccine development [224]. However, the rapid pace should not interfere with the quality of vaccines, and a substandard vaccine must be avoided to be commercialized even under the extreme global demand and pressure. An overview of designing and developing COVID-19 vaccines is depicted in Fig. 3.

## Patents on successful methodologies on various aspects of coronaviruses

Global research focuses on coronavirus infections, especially the recently happened outbreaks of SARS, MERS, and the most newly COVID-19. That involves the exploration of diagnostics, prophylactics, and therapeutics. Accordingly, the patenting of successful methodologies has gained immense importance, safeguarding the interests of scientists and institutions. Various patents are being filed, and many approved on diagnostic, prophylactic, and therapeutic aspects of coronaviruses and their diseases, especially SARS-CoV caused by SARS, MERS-CoV caused by MERS and the most newly SARS-CoV-2 caused COVID-19. As per one study, around 80% of patents are related to therapeutics, 35% for vaccines, and 28% for diagnostics agents or methods [121]. Similarly, on MERS, more than 100 patents are published on therapeutics, and more than 50 on diagnostics and prophylactics [121]. These patents cover a range of research areas.

Table 3 provides details about the various fields and research areas for which patents have been applied and granted. These areas include developing novel, rapid, and specific diagnostics that are cheap and readily available [121, 227–229]. For this purpose, the exploration of novel methods or techniques is being elucidated that can be transformed into diagnostic technology. Further, identifying diagnostic markers are vital. Similarly, for the treatment purpose, novel drugs or therapeutic agents are being explored [121]. These need to be effective against coronaviruses, including SARS-CoV, MERS-CoV, and SARS-CoV-2 [121, 230, 231]. These can be viral protease inhibitors [231], enzyme inhibitors [232], antivirals [228, 233], immunomodulatory [234], and treatment adjuvants [235]. That is the primary class that has attracted most of the patents [121]. Either a drug target is located, or a direct antiviral compound, molecule or agent is established, or an indirect immunomodulator that raises the immune response against the virus is identified as shown in Table 3. Vaccines, which are the main backbone for the prevention strategies and are mostly lacking, especially for novel coronaviruses, have shown immense research prospects and are attracting a sufficient number of patents as shown in Table 3 [121]. Exploring novel vaccine candidates, targeting appropriate antigens, evaluating immune responses, and transforming into a safe, effective, and potent vaccine are some of the areas on which patents have been published [236–238]. They can be univalent targeting a single pathogen (species/strain) [238, 239] or multivalent targeting many at a time [237]. Presently no specific therapeutics or vaccines are available against SARS-CoV-2. Since there is some degree of genomic and structural identity hence therapeutics active against SARS-CoV or MERS-CoV or other broad-spectrum antivirals (e.g., remdesivir, chloroquine) are being explored for SARS-CoV-2. Identification of specific therapeutic targets and vaccines candidates will enable the developing of concrete and potential drugs or vaccines that can prove useful in the prevention and control of SARS-CoV-2 [121].

**Table 3:** Patents on methodologies of different aspects of coronaviruses

Patent no.	Date	Basis	Research/patent title	Findings	References
2016-07-20	EP3045181A1	Vaccine	A novel vaccine against the middle east respiratory syndrome coronavirus (MERS-CoV)	The invention relates an immunogenic composition comprising the MERS-CoVN nucleocapsid protein and an immunogenic fragment thereof, or a nucleic acid molecule encoding the MERS-CoV N nucleocapsid protein and the immunogenic fragment thereof. Furthermore, the present invention relates to a vector comprising a nucleic acid molecule encoding the MERS-CoV N nucleocapsid protein and an immunogenic fragment thereof, for use as a vaccine as well as a method of inducing a protective immune response against MERS-CoV	Sutter et al. [238]
2007-02-15	JP2007502612A	Diagnosis, vaccine	Coronavirus, nucleic acid, protein, and methods for the generation of vaccine, medicaments, and diagnostics	A new coronavirus, human coronavirus NL63 (HCoV-NL63) is disclosed herein with a tropism that includes humans. Means and methods are provided for diagnosing subjects (previously) infected with the virus. Also offered are among other vaccines, medicaments, nucleic acids, and specific binding members	Van Der Hoek [227]
2012-07-05	ES2384445T3	Vaccine	Spike protein of canine respiratory coronavirus (crcv), polymerase, and hemaglutinin/esterase	A vaccine composition for vaccinating dogs, the composition comprising: a canine respiratory coronavirus (CRCV) containing a Spike (S) protein having a list of amino acid sequence, or a coronaviral S protein having at least 97% amino acid identity with the amino acid sequence, or an immunogenic fragment of Figure 4 of at least 200 amino acids in length, or a nucleic acid encoding said coronaviral S protein or said immunogenic fragment	Brownlie et al. [239]
2017-10-12	WO2017176596A1	Vaccine	Multivalent vaccines for rabies virus and coronaviruses	The present disclosure provides methods and compositions for inducing an immune response that confers dual protection against infections by either or both of a rabies virus and a coronavirus, and which can be used therapeutically for an existing infection with the rabies virus and a coronavirus to treat at least one symptom thereof and to neutralize or clear the infecting agents. In particular, the present disclosure provides a recombinant rabies virus vector comprising a nucleotide sequence encoding at least one coronavirus immunogenic glycoprotein fragment, as well as pharmaceutical compositions comprising the vaccine vectors	Johnson et al. [237]
2017-12-28	WO2017222935A1	Treatment and control	Small molecule therapeutic inhibitors against picornaviruses, caliciviruses, and coronaviruses	Antiviral protease inhibitors are disclosed, along with related antiviral dipeptidyl compounds, macrocyclic derivatives thereof, and methods of using the same to treat or prevent viral infection and disease from coronaviruses, caliciviruses, and picornaviruses	Chang et al. [261]
2018-05-22	S9975885B2	Treatment and control	Broad-spectrum non-covalent coronavirus protease inhibitors	This invention pertains to materials and methods for the treatment of patients with coronavirus infection and the control of zoonotic disease outbreaks using broad-spectrum non-covalent coronavirus protease inhibitors	Emma et al. [231]
2017-08-03	WO2017129975A1	Vaccine	Attenuated infectious bronchitis virus	The present invention provides a live, attenuated coronavirus comprising a mutation in non-structural protein nsp-3 and deletion of accessory proteins 3a and 3b. The coronavirus may be used as a vaccine for treating and preventing disease, such as infectious bronchitis, in a subject	Bickerton et al. [262]
2019-12-05	JP2019206573A	Immunity boosters	Immunomodulation methods and compositions	Methods and compositions for immunomodulation are provided. Cells containing exogenous antigens and uses thereof are produced. The present invention includes, for example, a way of inducing immune tolerance, comprising enucleated hematopoietic cells that express an exogenous antigen to a human subject suffering from or at risk of developing an autoimmune disease, disorder or condition Administering a pharmaceutical composition, wherein the pharmaceutical composition is administered in an amount effective to induce immune tolerance in the subject against the antigen that mediates the autoimmune disease, disorder or condition	Jordi et al. [234]
2020-01-02	US20200002370A1	Treatment, adjuvants	Novel compounds	The invention also relates to the use of said compounds, combinations, compositions and medicaments, in the treatment of diseases in which modulation of STING (Stimulator of Interferon Genes) is beneficial, for example, inflammation, allergic and autoimmune diseases, infectious diseases, cancer, pre-cancerous syndromes and as vaccine adjuvants	Adams and Lian [235]
2019-11-14	US20190343862A1	Immunomodulators	Delivery of RNA to trigger multiple immune pathways	RNA encoding an immunogen is co-delivered to non-immune cells at the site of delivery and also to immune cells, which infiltrate the site of delivery. The responses of these two cell types to the same delivered RNA lead to two different effects, which interact to produce a robust immune response against the immunogen. The non-immune cells translate the RNA and express the immunogen. Infiltrating immune cells respond to the RNA by expressing type I interferons and pro-inflammatory cytokines, which produce a local adjuvant effect that acts on the immunogen-expressing non-immune cells to upregulate major histocompatibility complex expression, thereby increasing presentation of the translated protein to T cells. The effects on the immune and non-immune cells can be achieved by a single delivery of a single RNA, e.g., by a single injection	Geall et al. [263]
2018-12-18	US10154985B2	Treatment	Method for treating a glycoprotein-related disease	A method for treating a glycoprotein-related disease is disclosed, which comprises: administering a first effective amount of phenol red and a second effective amount of an organic arsenic compound to a subject in need thereo	Lu [264]
2016-11-24	US20160339097A1	Recombinant vaccine proteins	Coronavirus proteins and antigens	Disclosed herein are embodiments of a method for collecting, extracting, or eluting proteins and antigens from cells infected with the coronavirus. The coronavirus maybe a porcine coronavirus, such as porcine epidemic diarrhoea virus (PEDV) or porcine delta coronavirus (PDCoV). Also disclosed are embodiments of a composition comprising the coronavirus proteins and antigens, and embodiments of a method of using such a composition. Applications for the composition include, but are not limited to, use in the preparation of antibodies against the proteins and antigens, use as reference markers for coronavirus proteins, and use in an immunogenic composition, such as in a vaccine composition	Kim [265]
2017-01-19	WO2017010835A1	Drug/treatment	Use of radotinib for prevention or treatment of viral respiratory disease	The present invention relates to a new use of a compound of Chemical Formula 1 (radotinib) in the prevention or treatment of viral respiratory disease. According to the present invention, a compound of Chemical Formula 1 or a pharmaceutically acceptable salt thereof can be used for the prevention, alleviation, or treatment of coronavirus infection. Specifically, the present invention can be used as a useful antiviral agent for the prevention or treatment of disease caused by infection of the Middle East respiratory syndrome-coronavirus (MERS-CoV).	Kim et al. [232]
2016-06-15	CN105671209A	Diagnosis	Primers, probe, kit, and method for detecting bovine coronavirus	The invention discloses primers, a probe, a kit and a method for detecting bovine coronavirus. The method comprises the following steps: by using cDNA (complementary deoxyribonucleic acid) obtained by carrying out RNA (ribonucleic acid) reverse transcription on the detected sample as a template, carrying out fluorescent quantitative PCR (polymerase chain reaction) amplification, and comparing the amplification curve CT value. The quantitative PCR primers are designed at the bovine coronavirus conserved gene (N gene), and a probe process is adopted to establish a fluorescent quantitative PCR method. The primers, probe, kit, and method are practical and straightforward to operate, have the advantages of high specificity, high sensitivity, and favourable repetitiveness, and are suitable for the demands for quick and accurate detection of modernized cultivation farms	Wei et al. [229]
2016-06-1	CN105671006A	Diagnosis and treatment	High-efficiency ranilla luciferase gene expression recombinant HCoV-OC43 (human coronavirus OC43) virus and application thereof	The invention discloses a high-efficiency ranilla luciferase gene expression recombinant human coronavirus OC43 virus and application thereof to a screening of antiviral medicines. By an overlap PCR (polymerase chain reaction) method, a ranilla luciferase gene is replaced or inserted into accessory genes (ns2 and ns12.9) to be cloned into human coronavirus OC43 full-length infectious clone pBAC-OC43FL, and four ranilla luciferase gene expression recombinant viruses, including rOC43-ns2DelRluc, rOC43-ns2FusionRluc, rOC43-ns12.9StopRluc, and rOC43-ns12.9FusionRluc, are obtained respectively. The virus rOC43-ns2DelRluc is efficient in ranilla luciferase gene expression and similar to a parent virus HCoV-OC43-WT in the virus growth curve, the inserted reporter gene is stable in a serial passage process, and the virus rOC43-ns2DelRluc can be successfully applied to antiviral medicine screening experiments and has an extensive application prospect in high-throughput screening of anti-coronavirus medicines and host antiviral genes	Shenliang, [228]
2016-08-10	CN105837487A	Prevention /treatment	Small-molecule inhibitor against MERS-CoV main protease, and preparation method and application thereof	The invention provides a small-molecule inhibitor against MERS-CoV main protease. The small-molecule inhibitor is designed on the basis of the crystal structure of the main protease of the novel coronavirus MERS-CoV. The invention also provides a synthetic method for the small-molecule inhibitor and application of the small-molecule inhibitor in the preparation of drugs used for preventing and treating MERS-CoV infections. The small-molecule inhibitor against MERS-CoV main protease can substantially inhibit the activity of the main protease of the MERS coronavirus, has an excellent inhibitory activity to the main protease of coronaviruses like SARS and MHV, and presents good application prospects in preparation of drugs used for preventing or treating coronavirus infection	Rao et al. [266]
2016-09-01	WO2016138160A1	Recombinant peptides/antibodies treatment	Middle east respiratory syndrome coronavirus immunogens, antibodies, and their use	Methods of inducing an immune response in a subject to the Middle East respiratory syndrome coronavirus (MERS-CoV) are provided. In several embodiments, the immune response is a protective immune response that inhibits or prevents MERS-CoV infection in the subject. Recombinant MERS-CoV polypeptides and nucleic acid molecules encoding the same are also provided. Additionally, neutralizing antibodies that specifically bind to MERS-CoV S protein and antigen-binding fragments thereof are disclosed. The antibodies and antigen-binding fragments are useful, for example, in methods of detecting MERS-CoV S protein in a sample or in a subject, as well as methods of preventing and treating a MERS-CoV infection in a subject	Graham et al. [267]
2016-06-01	CN105624122A	Mutant virus as a vaccine	Avian infectious bronchitis virus natural mutant	The invention relates to the field of virology. It aims to provide an avian infectious bronchitis virus natural mutant, kept in China General Microbiological Culture Collection Center, named infectious bronchitis virus, and collected under CGMCC No. 11491. A genome of this mutant is 27, 541 lbp in length, a complete genome sequence of the mutant is as shown in SEQ ID NO: 1, with structural characteristics of a typical IBV genome coding structure. This mutant having special genes inserted in and provided by the invention can break the immunity protection of vaccines to breed in an organism, this mutant can proliferate in susceptible chickens without causing significant pathogenicity to sensitive chickens, and thus this mutant can serve as the base material for the further study on mutation mechanism of an attenuated vaccine or IBV virus strain	Liao et al. 2016[268]
2017-08-08	CN107022008A	Prevention and treatment	Suppress polypeptide and its application of human coronavirus’s infection broad spectrum	The invention belongs to the biomedicine field. It is related to the polypeptide for suppressing human coronavirus infection, and in particular, to polypeptide and its application of human coronary virus’s disease can be contained a broad spectrum. The S2 regions based on coronavirus S protein of the invention are more conservative, and features of similar syncretizing mechanisms provide can be to the polypeptide of the infection with broad-spectrum inhibitory action of two or more human coronary virus. The present invention is the results showed obtain “general character” of the human coronavirus, the identical syncretizing mechanism in, i.e., similar HR regions and its mediation, and provide the serial polypeptides of HCoV-EK as the point of penetration, the polypeptide not only has preferable inhibition to some currently a popular human coronaviruses, and equally has an excellent inhibitory activity to the class SARS virus (RsSHC014-CoV or RsW1V1-CoV) for being possible to infect the humankind. The present invention can provide the drug candidate for prevention and treatment for the novel human coronavirus for being still possible to outburst in popular human coronavirus and future at present	Shibo et al. [269]
2018-02-13	US9889194B	Immunogenic proteins	Immunogenic composition for MERS coronavirus infection	Described herein are immunogenic compositions for preventing infection with Middle East respiratory syndrome coronavirus (MERS-CoV) wherein the immunogenic compositions comprise at least a portion of the MERS-CoV S protein and an immunopotentiator	Jiang et al. [270]
2019-11-21	US20190351049A1	Spike protein	Human Antibodies to Middle East Respiratory Syndrome - Coronavirus Spike Protein	The present invention provides monoclonal antibodies that bind to the Middle East Respiratory Syndrome—Coronavirus (MERS-CoV) spike protein and methods of use. In various embodiments of the invention, the antibodies are fully human antibodies that bind to MERS-CoV spike protein. In some embodiments, the antibodies of the invention are useful for inhibiting or neutralizing MERS-CoV activity, thus providing a means of treating or preventing MERS infection in humans. In some embodiments, the invention provides for a combination of one or more antibodies that bind to the MERS-CoV spike protein for use in treating MERS infection. In certain embodiments, the one or more antibodies bind to distinct non-competing epitopes comprised in the receptor-binding domain of the MERS-CoV spike protein	Kyratsous et al. [271]
2019-12-05	US20190365925A1	Antigen targets	AAV vectors targeted to the central nervous syste	The invention relates to chimeric AAV capsids targeted to the central nervous system, virus vectors comprising the same, and methods of using the vectors to target the central nervous system. The invention further relates to chimeric AAV capsids targeted to oligodendrocytes, virus vectors comprising the same, and methods of using the vectors to target oligodendrocytes	Gray and McCown, [272]
2019-09-05	JP2019146588A	Vaccine	Priming of an immune response	The present invention provides a method for expanding and improving immune response and, at the same time, increasing the speed of response against a pathogenic antigen or a cancer antigen. A two-step prime-boost method whereby the immune system is first primed with a nucleic acid construct comprising an invariant chain or a variant thereof, followed by a booster vaccine using any type of suitable vaccine Administration) to sufficiently stimulate the immune response generated by vaccine administration	Thomsen et al. [273]
2019-10-17	US20190314497A1	Treatment	Compositions and methods for treating viral infections through stimulated innate immunity in combination with antiviral compounds	Embodiments are directed to compositions and methods for treating viral infections	Dickey et al. [274]
2019-03-12	US10226434B2	Treatment	Design, synthesis, and methods of use of acyclic fleximer nucleoside analogues having anti-coronavirus activity	The present invention is directed to compounds, techniques, and compositions for treating or preventing viral infections using nucleosides analogues. Specifically, the present invention provides for the design and synthesis of acyclic fleximer nucleoside analogues, having increased flexibility and ability to alter their conformation structures to provide increased antiviral activity potential with the result of inhibiting several coronaviruses	Radtke et al. [275]
2020-02-04	US10548971B2	Vaccine	MERS-CoV vaccine	Disclosed herein is a vaccine comprising a Middle East Respiratory Syndrome coronavirus (MERS-CoV) antigen. The antigen can be a consensus antigen. The consensus antigen can be a consensus spike antigen. Also disclosed herein is a method of treating a subject in need thereof by administering the vaccine to the topic	Weineret al. 2020[276]
2019-05-08	Publication of JP6508605B2	Vaccine	Modular DNA binding domain and method of use	The present invention provides a method of selectively recognizing base pairs in a target DNA sequence by a polypeptide, a modified polypeptide that recognizes explicitly one or more base pairs within a target DNA sequence, and a DNA that is modified so that it can be recognized explicitly by the polypeptide, and specific use of polypeptides and DNA in NA targeting and methods of modulating the expression of target genes in cells	Bonas et al. [277]
2019-09-10	US10406229B2	Vaccine	Methods and compositions related to inhibition of viral entry	Disclosed herein are compositions and arrangements for inhibiting viral entry	Francis et al. [278]
2018-12-20	AU2015340134B2	Antigenic gene identification	Microfluidic device for detecting target gene, the method for manufacturing same, and method for detecting using same	The present invention provides a target gene capable of being differentiated by the naked eye by amplifying the target gene to selectively block the fluid path and, specifically, a microfluidic device for detecting pathogen genes, and a detection method using the same. Therefore, the present invention can conveniently identify a single target gene, such as a single pathogen, or at the same time, several target genes, such as several pathogens, without complicated mechanical devices	Jung et al. [279]
2018-01-30	US9878024B2	Enhanced immune response	Methods and compositions using Listeria for enhancing immunogenicity by prime-boost	Provided herein are prime-boost regimens and materials used therein. The prime-boost regimens enhance the immune response to a target antigen. The vaccines used for boost are comprised of recombinant attenuated metabolically active *Listeria* that encodes an expressible antigen that is cross-reactive with the target antigen. In some examples, the immune response is a cellular immune response	Dubensky et al. [280]
2017-08-0	US9719107B2	Recombinant gene and protein expression	Construction of fully-deleted adenovirus-based gene delivery vectors and uses thereof	The embodiments disclosed herein relate to the construction of fully-deleted Adenovirus-based gene delivery vectors packaged without helper Adenovirus, and more particularly to their use in gene therapy for gene and protein expression, vaccine development, and immunosuppressive therapy for allogeneic transplantation. In an embodiment, a method for propagating an adenoviral vector includes (a) providing an Adenovirus packaging cell line; (b) transfecting a fully-deleted Adenoviral vector construct into the cell line; and optionally (c) transfecting a packaging construct into the cell line, wherein the fully-deleted Adenoviral vector construct and optionally the packaging construct can transfect the Adenovirus packaging cell line resulting in the encapsidation of a fully-deleted Adenoviral vector independent of helper Adenovirus. In an embodiment, a target cell is transduced with the encapsidated fully-deleted Adenoviral vector for treating a condition, disease, or disorder	Brennan et al. [281]
2019-06-04	US10307439B2	Drug treatment	Substituted nucleosides, nucleotides and analogues thereof	Disclosed herein are nucleosides, nucleotides and nucleotide analogues, methods of synthesizing the same and methods of treating diseases and conditions such as a Coronaviridae virus, a Togaviridae virus, a Hepeviridae virus and a Bunyaviridae virus infection with one or more nucleosides, nucleotides and nucleotide analogue	Blatt et al. [282]
2008-05-22	WO2007062656A3	Vaccine	A nucleotide vaccine	The present invention relates to a vaccine comprising a nucleic acid construct such as a DNA construct, primarily a nucleic acid construct containing sequences encoding invariant chain operatively linked to antigenic protein or peptide encoding sequences. The vaccine stimulates an immune response, especially an immune response in an MHC-I dependent, but CD4+ T-cell independent manner	Holst et al. [283]
2018-01-23	US9872900B	Vaccine	Nucleic acid vaccines	The invention relates to compositions and methods for the preparation, manufacture, and therapeutic use of ribonucleic acid vaccines (NAVs) comprising polynucleotide molecules encoding one or more antigens	Ciaramella et al. [284]
2019-05-14	US10286065B2	Treatment and immune modulators	Compositions and methods for treating viral infections through stimulated innate immunity in combination with antiviral compounds	Embodiments are directed to compositions and methods for treating viral infections	Dickey et al. [285]
2019-10-09	EP3194423B1	Treatment antiviral	Method for preparing a vaccine antigen, resulting ina vaccine antigen and uses	The present invention relates to a method for making a vaccine antigen comprising a membrane protein, as well as a vaccine antigen and a vaccine, and uses thereof	Rosa-Calatrava et al. [286]
2018-11-20	US10130701B2	Treatment/vaccine	Coronaviru	The present invention provides a live, attenuated coronavirus comprising a variant replicase gene encoding polyproteins consisting of a mutation in one or more of non-structural protein(s) (nsp)-10, nsp-14, nsp-15 or nsp-16. The coronavirus may be used as a vaccine for treating and preventing disease, such as infectious bronchitis, in a subject	Bickerton et al. [287]

Specifically, with regards to COVID-19, three drugs are in the race for patents. These include remdesivir of Gilead Sciences^®^, hydroxychloroquine of Sanofi^®^ and Kaletra (Lopinavir+Ritonavir) of Abbvie^®^. They are patented for use against viruses (Ebola), malaria and HIV/AIDS, respectively, however, have shown potential for application in COVID-19 hence being evaluated in clinical trials (https://ttconsultants.com/blog/potential-covid-19-drugs-and-their-patents-in-major-jurisdictions/). Wuhan Institute of Virology in China has filed a patent for the application of remdesivir in COVID-19 [240] and Suzhou-based BrightGene Bio-Medical Technology for the synthesis of its active ingredient [241, 242]. Chinese were first to evaluate the effectiveness of remdesivir, chloroquine and Kaletra in treating COVID-19 patients [25, 136]. However, simultaneous intentions for patent applications raised global concerns [242]. This led to a sort of conflict between Chinese institutes and the pharmaceutical companies having original patents for the drugs [242]. Thus, avoiding such differences and focusing on improving the health of people in times of pandemic need to be the priority rather than personnel monetary gains [242].

Similarly, in the case of vaccines which are in final stages of development, inferences from related coronaviruses like SARS and MERS are being taken. There are around 500 patents on SARS and 50 on MERS vaccines; however, it is unknown how many will be granted [35, 121]. Nevertheless, these can provide the basis for vaccine development for COVID-19 [35, 121].

## Conclusion and future perspectives

Currently, the SARS-CoV-2 outbreak has taken a disastrous turn with high toll rates alone in China itself, most likely. The infection is spreading across the globe. There are no licensed vaccines or therapeutic agents (i.e., antivirals and monoclonal antibodies) indicated for this coronavirus prevention or treatment. However, researchers are working to develop countermeasures. Several vaccine candidates for both SARS-CoV-2 are in early clinical trials. This review is an accumulative hub of the latest knowledge and out comings of all novel approaches to tackle this deadly disease. Validated and clinically proven therapeutic measures are in great need at this hour of crisis to ensure global safety and on stopping this pandemic before it leads to distressing global outbreaks [243].

Under the current COVID-19 pandemic scenario, the global threats are increasing, more countries are being predisposed, and new outbreaks are being reported, increasing the number of infected cases and risks to non-infected persons—this necessitates timely intervention for appropriate management of affected ones and prevention of threats to healthy ones. Non-availability of specific antivirals against COVID-19 is causing heavy tolls in infected persons. Utilization of conventional therapies as a supportive treatment, though, helps in managing severity but is proving ineffective on a long-term basis as the overall mortality is changing significantly daily. Non-specific antiviral therapy by oseltamivir, ganciclovir, antibacterial therapy by moxifloxacin, ceftriaxone, azithromycin; and glucocorticoid therapy is being improvised by adding broad-spectrum antivirals such as remdesivir, chloroquine, or lopinavir/ritonavir. Passive treatment by α-interferon atomization inhalation, immunoglobulin G therapy, or plasma therapy is proving beneficial, but require confirmation in clinical trials in order to be recommended [244–246].

However, there is a dire need for development and evaluation of specific antivirals against COVID-19. There have been attempts to explore the applicability of already existing antivirals, potential antivirals, or a combination thereof along with supportive medicines; however, the focus should be on designing and adopting safe and effective modalities against SARS-CoV-2. These include developing neutralizing antibodies, oligonucleotides targeting SARS-CoV-2 RNA genome, passive antibody transfer, drug repurposing using available antivirals, anti-viral proteases, blocking coronavirus receptors like ACE2, targeting spike proteins, and combination therapies [247–253].

Alternative measures like neutralizing antibodies, oligonucleotides, passive antibody transfer, and drug repurposing can bring a revolutionary change until the core researchers are busy finding the specific target to curb the SARS-CoV-2 which can be time taking and still at large [254–259].
